# Determinants of Cervical Cancer Screening Accuracy for Visual Inspection with Acetic Acid (VIA) and Lugol’s Iodine (VILI) Performed by Nurse and Physician

**DOI:** 10.1371/journal.pone.0170631

**Published:** 2017-01-20

**Authors:** Amidu O. Raifu, Mariam El-Zein, Ghislain Sangwa-Lugoma, Agnihotram Ramanakumar, Stephen D. Walter, Eduardo L. Franco, For the Congo Screening Study Group

**Affiliations:** 1 Department of Epidemiology and Biostatistics, Western University, Hamilton, Ontario, Canada; 2 Division of Cancer Epidemiology, Department of Oncology, McGill University, Montreal, Quebec, Canada; 3 Department of Obstetrics & Gynaecology, University of Kinshasa, Kinshasa, Democratic Republic of Congo; 4 Department of Clinical Epidemiology & Biostatistics, McMaster University, Hamilton, Ontario, Canada; Universidade Estadual de Maringa, BRAZIL

## Abstract

**Background:**

Visual inspection with acetic acid (VIA) and Lugol’s iodine (VILI) are used to screen women for cervical cancer in low-resource settings. Little is known about correlates of their diagnostic accuracy by healthcare provider. We examined determinants of VIA and VILI screening accuracy by examiner in a cross-sectional screening study of 1528 women aged 30 years or older in a suburb of Kinshasa, Democratic Republic of Congo.

**Methods:**

We used a logistic regression model for sensitivity and specificity to estimate the diagnostic accuracy of VIA and VILI, independently performed by nurse and physician, as a function of sociodemographic and reproductive health characteristics.

**Results:**

Nurses rated tests as positive more often than physicians (36.3% vs 30.2% for VIA, 26.2% vs 25.2% for VILI). Women’s age was the most important determinant of performance. It was inversely associated with sensitivity (nurse’s VIA: p<0.001, nurse’s VILI: p = 0.018, physician’s VIA: p = 0.005, physician’s VILI: p = 0.006) but positively associated with specificity (all four combinations: p<0.001). Increasing parity adversely affected sensitivity and specificity, but the effects on sensitivity were significant for nurses only. The screening performance of physician’s assessment was significantly better than the nurse’s (difference in sensitivity: VIA = 13%, VILI = 16%; difference in specificity: VIA = 6%, VILI = 1%).

**Conclusions:**

Age and parity influence the performance of visual tests for cervical cancer screening. Proper training of local healthcare providers in the conduct of these tests should take into account these factors for improved performance of VIA and VILI in detecting cervical precancerous lesions among women in limited-resource settings.

## Introduction

Cervical cancer is the third most common cancer diagnosed among women worldwide and ranks seventh among all causes of death, with an estimated 529,000 new cases and 275,000 deaths in 2008 [1,2]. Nearly 85% of cases occur in developing countries, mostly in Eastern Africa, Melanesia, Southern Africa, and Middle Africa [1]. Cervical cancer is a preventable disease if precancerous lesions are detected early through effective screening programs, but establishment and successful implementation of the latter is challenging in low-income countries. For cervical cancer screening to be successful in resource-limited settings, the screening test, diagnosis, and treatment must either be provided on-site or in clinics accessible to the majority of women at risk. In search of simple, cost-effective screening methods for cervical cancer prevention in low-resource settings, visual inspection with acetic acid (VIA) and visual inspection with Lugol’s iodine (VILI) have been considered as alternative, less technically complex tests to conventional cytology. However, their diagnostic accuracy in detecting high-grade precursor lesions and invasive cervical cancer varies across studies. VIA sensitivity and specificity ranged from 55% to 96% and from 49% to 98%, respectively, whereas VILI sensitivity and specificity ranged from 44% to 98% and from 75% to 91%, respectively [3–7].

The diagnostic performance of VIA and VILI for cervical cancer screening, and determinants of their positivity have been extensively evaluated [4,6–12]. However, no study has investigated how patient characteristics might influence their diagnostic accuracy. The objective of the present study is to examine demographic, sexual behavior, and clinical determinants of the sensitivity and specificity of VIA and VILI tests, independently performed by nurse and physician, in a community-based cross-sectional cervical cancer screening study conducted in a primary health care setting in the suburbs of Kinshasa, the Democratic Republic of Congo.

## Materials and Methods

### Study design and population

**Fig 1:**
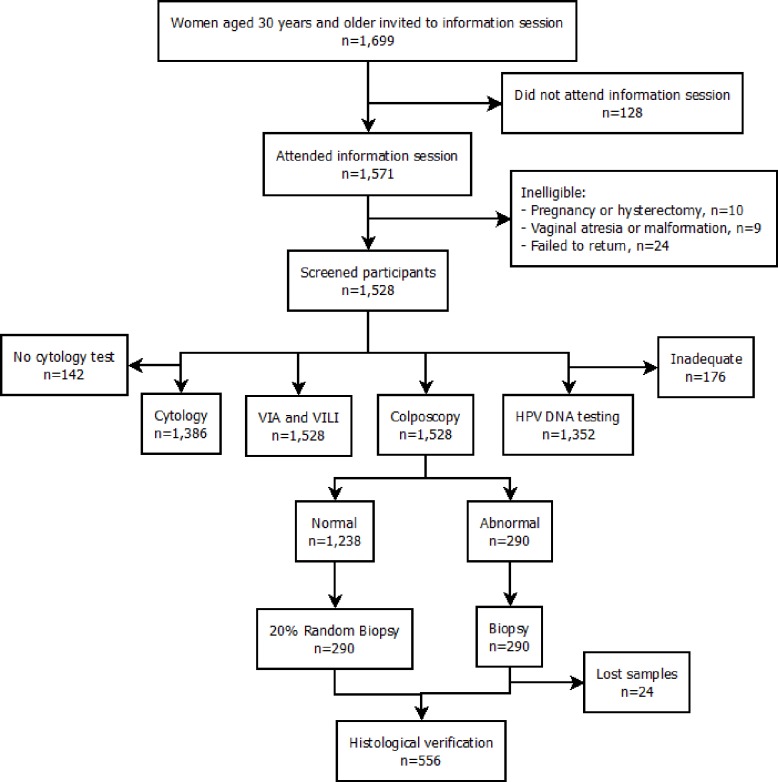
Overview of the Congo Community-Based Screening Study population and procedures. Eligible women were screened by conventional cytology, HPV DNA testing, VIA, VILI, and colposcopy.

A detailed description of the design, study population, eligibility criteria, data collection methods, and study outcomes of the Congo Community-Based Screening Study is provided elsewhere [7,13,14]. Fig 1 presents an overview of the study population and procedures. Briefly, 1699 women aged 30 years and older were invited to attend an educational session on cervical cancer prevention provided by a trained nurse, between 2003 and 2004 at the Mbuku Healthcare Center, Kinsenso commune. Of these, 1571 women accepted, and 1528 eligible women were interviewed by a trained nurse using a structured, standardized questionnaire to collect information on sociodemographic, reproductive, clinical, lifestyle, and sexual behavior characteristics.

Women were screened by conventional cytology (smears read offsite in Lyon, France), HPV DNA testing (Hybrid Capture 2), VIA, VILI, and colposcopy. Cytological diagnoses were based on the Bethesda system [15], i.e., negative for intraepithelial lesions or malignancy (NILM), atypical squamous cells of undetermined significance (ASC-US), low-grade squamous intraepithelial lesion (LSIL), high-grade squamous intraepithelial lesion (HSIL), and cancer (no other findings were recorded in this study). Cytology smears were read by a cytotechnologist and reviewed by a gynecologic-cytopathologist without knowledge of disease status or results of the other screening tests. VIA and VILI were independently performed by one of six attending nurses and by an experienced gynecologist (GSL) who trained the nurses. Colposcopy-directed biopsies were obtained from women with cervical abnormalities. Cervical biopsies were also performed in a random sample of women (~20%) who had normal colposcopic findings. The histopathological slides were shipped to Montreal and read at the pathology lab of the Jewish General Hospital, a teaching institution affiliated with McGill University. Biopsy slides were read in research capacity by an experienced gynecologic pathologist who was blinded to the screening test results and colposcopic impressions. Disease was defined as cervical intraepithelial neoplasia (CIN) of grade 2 or worse (CIN2+). All women provided verbal or written informed consent, depending on their level of literacy. A local community health representative independent from the study ascertained that all instances of verbal consent were given after subjects listened attentively to the full text of the consent form read by the study staff. The PATH Human Subjects Protection Committee and the Institutional Review Board of the Kinshasa School of Public Health approved the study, the informed consent, and the above approach for obtaining it from subjects.

### Statistical analysis

We used a method originally derived by Cornfield (1962) to estimate a subgroup risk of coronary heart disease [16], and later applied by Coughlin et al. (1992) to estimate the sensitivity and specificity of a screening test [17]. Four multivariate logistic regression models were used to estimate the diagnostic accuracy indices and corresponding 95% confidence intervals (CI) for VIA and VILI tests performed by nurses and clinician. Compared to the crude approach of calculating sensitivity and specificity of screening tests, this model-based approach allows for evaluating and adjusting for predictor variables, which was integral to our analysis. In addition, it makes use of all data on women with and without cervical lesions through inclusion of the gold standard variable in the model (in this case: histology supplemented by colposcopy).

We chose predictors based on a backward stepwise selection procedure, using an arbitrary p-value of 0.15 and the likelihood ratio test, to accommodate important covariates. We also used judgment based on biological plausibility for variables that could potentially influence the performance of VIA and VILI, e.g., menopausal status. The final models included age (30–39, 40–49, 50–59, ≥ 60 years), age at first sexual intercourse (< 18 years, ≥ 18 years), number of lifetime sexual partners (0–1, 2–3, ≥ 4), history of sexually transmitted diseases (no, yes), parity (0–1, 2–5, 6–9, ≥ 10), menopausal status (pre vs. post), healthcare provider (medical doctor, other), history of Pap test (never, ever), HPV DNA test results (negative, positive), and cytology test results (NILM, ASC-US, LSIL, HSIL, and cancer). The disease status variable was based on the results from the colposcopy supplemented with histological verification if a biopsy had been taken (Fig 1). Eight variables had missing values; number of lifetime sexual partners (0.3%), age at first sexual intercourse (0.4%), parity (0.5%), history of Pap smear (0.6%), healthcare provider (2.8%), menopausal status (3.2%), cytology (9.3%), and HPV DNA (11.5%). Our preliminary logistic regression analyses showed that 379 observations (25% of the data) would be omitted due to case-wise deletion. To avoid loss of information, all statistical analyses were based on a derivative dataset using multiple imputation to replace missing data by expected values.

A non-parametric test developed by Cuzick (1985) [18] was used to assess trends across categorically ordered predictors. For binary predictors, a 2-sample z-test for proportions was performed to compare the screening accuracy of VIA and VILI between the two categories. We also calculated, across predictors, the differences in sensitivity and specificity of VIA and VILI between nurse and physician along with corresponding 95%CIs. All statistical analyses and multiple imputation were carried out using the Stata statistical software package (StataCorp, Release 12.1, College Station TX) with two-sided p-values.

## Results and Discussion

The mean age of participants was 42.7 years (range: 30–85 years). Among the 1528 women enrolled, nurses reported a positive VIA for 555 (36.3%) and a positive VILI for 401 (26.2%), whereas VIA and VILI positivity according to the physician’s assessment were recorded for 462 (30.2%) and 385 (25.2%) women, respectively. Table 1 shows the distribution of VIA and VILI positivity, independently performed by nurse and physician, according to selected sociodemographic, sexual behavior, and clinical variables. The proportions of VIA and VILI positivity were lower for physician-assessed compared to nurse-administered tests across most non-clinical characteristics.

**Table 1:** Characteristics of participants with positive test results from visual inspection methods performed by nurse and physician, the Congo Community-Based Screening Study.

Variable	Categories	Number of women	Positivity by test and examiner, n (%)			
			VIA		VILI	
			Nurse (n = 555)	Physician(n = 462)	Nurse (n = 401)	Physician (n = 385)
Age (years)	30–39	529	231 (43.7)	200 (37.8)	157 (29.7)	158 (29.9)
	40–49	556	190 (34.2)	157 (28.2)	144 (25.9)	139 (25.0)
	50–59	294	88 (29.9)	63 (21.4)	64 (21.8)	55 (18.7)
	60+	149	46 (30.9)	42 (28.2)	36 (24.2)	33 (22.2)
Marital status	Married/Cohabitation	1146	425 (37.1)	345 (30.1)	296 (25.8)	285 (24.9)
	Others	382	130 (34.0)	117 (30.6)	105 (27.5)	100 (26.2)
Education	None	299	100 (33.4)	76 (25.4)	71 (23.8)	64 (21.4)
	Primary	712	247 (34.7)	202 (28.4)	181 (25.4)	171 (24.0)
	Secondary or higher	510	204 (40.0)	180 (35.3)	145 (28.4)	146 (28.6)
Occupation	None	558	221 (39.6)	175 (31.4)	151 (27.1)	151 (27.1)
	Manual	812	286 (35.2)	239 (29.4)	211 (26.0)	196 (24.1)
	Skilled	132	38 (28.8)	40 (30.3)	35 (26.5)	34 (25.8)
	Professional	15	5 (33.3)	5 (33.3)	3 (20.0)	3 (20.0)
Smoking	No	1092	418 (38.3)	338 (30.9)	293 (26.8)	277 (25.4)
	Yes	434	136 (31.3)	122 (28.1)	107 (24.6)	106 (24.4)
Age at first sexual intercourse (years)	< 18	934	361 (38.7)	292 (31.3)	261 (27.9)	240 (25.7)
	18+	580	187 (32.2)	164 (28.3)	133 (22.9)	139 (24.0)
Number of lifetime sexual partners	0–1	812	266 (32.8)	238 (29.3)	204 (25.1)	198 (24.4)
	2–3	561	230 (41.0)	179 (31.9)	156 (27.8)	149 (26.6)
	4+	150	56 (37.3)	43 (28.7)	38 (25.3)	36 (24.0)
History of STD	No	1320	468 (35.5)	388 (29.4)	330 (25.0)	323 (24.5)
	Yes	208	87 (41.8)	74 (35.6)	71 (34.1)	62 (29.8)
Parity	0–1	140	35 (25.0)	29 (20.7)	20 (14.3)	25 (17.9)
	2–5	489	212 (43.4)	182 (37.2)	153 (31.3)	140 (28.6)
	6–9	702	241 (34.3)	190 (27.1)	174 (24.8)	173 (24.6)
	10+	189	64 (33.9)	58 (30.7)	52 (27.5)	44 (23.3)
Menopausal status	Pre	981	404 (41.2)	341 (34.8)	283 (28.8)	285 (29.0)
	Post	498	137 (27.5)	108 (21.7)	106 (21.3)	89 (17.9)
Healthcare provider	Medical doctor	895	361 (40.3)	277 (31.0)	255 (28.5)	229 (25.6)
	Other health provider	590	172 (29.2)	170 (28.8)	132 (22.4)	142 (24.1)
History of Pap test	Never	1469	546 (37.2)	448 (30.5)	396 (27.0)	375 (25.5)
	Ever	50	7 (14.0)	13 (26.0)	3 (6.0)	9 (18.0)
HPV DNA	Negative	1183	416 (35.2)	329 (27.8)	297 (25.1)	266 (22.5)
	Positive	169	77 (45.6)	83 (49.1)	59 (34.9)	74 (43.8)
Cytology	NILM	1270	453 (35.7)	377 (29.7)	321 (25.3)	306 (24.1)
	ASC-US	40	20 (50.0)	14 (35.0)	11 (27.5)	12 (30.0)
	LSIL	45	22 (48.9)	20 (44.4)	19 (42.2)	18 (40.0)
	HSIL	16	9 (56.2)	12 (75.0)	9 (56.2)	12 (75.0)
	Cancer	15	9 (60.0)	11 (73.3)	9 (60.0)	11 (73.3)
Colposcopy result without biopsy	< CIN2	1505	535 (35.6)	439 (29.2)	379 (25.2)	362 (24.0)
	CIN2+	23	20 (87.0)	23 (100.0)	22 (95.6)	23 (100.0)
Colposcopy result with biopsy	< CIN2	1497	531 (35.5)	434 (29.0)	378 (25.2)	357 (23.8)
	CIN2+	31	24 (77.4)	28 (90.3)	23(74.2)	28 (90.3)

Abbreviations: ASC-US: Atypical Cells of Undetermined Significance; CIN: Cervical Intraepithelial Neoplasia; HPV DNA: Human Papillomavirus DNA test; HSIL: High Squamous Intraepithelial Lesion; LSIL: Low Squamous Intraepithelial Lesion; NILM: Negative for Intraepithelial Lesion or Malignancy; STD: Sexually Transmitted Diseases; VIA: Visual Inspection with Acetic acid; VILI: Visual Inspection with Lugol’s Iodine.

Tables 2 and 3 show the diagnostic accuracy of VIA and VILI, respectively, for nurse and physician, stratified according to different characteristics and mutually adjusted for each other. Irrespective of test and examiner, there was a trend for sensitivity to decrease with the age of the woman, an observation that was mirrored, expectedly, on menopausal status. The trends were opposite for specificity, and were statistically significant. Increasing parity adversely affected sensitivity and specificity, but the effects on sensitivity were significant for nurses only and the effects on specificity were significant for physician-performed tests only. Although the examiners did not know the HPV test results, HPV positivity seemed to improve the examiner’s propensity to detect disease, but not significantly. On the other hand, irrespective of test and examiner, specificity degraded significantly when women were HPV positive. With respect to cytology, except for VIA done by a nurse, there were similar trends of increased sensitivity and decreased specificity as grade of lesion increased.

**Table 2:** Determinants of VIA sensitivity and specificity performed by nurse and physician: the Congo Community-Based Screening Study.

Variable	Categories	VIA Sensitivity (95% CI)^a^			VIA Specificity (95% CI)^a^		
		Nurse	Physician	Difference^b^	Nurse	Physician	Difference^b^
Age (Years)	30–39	0.82 (0.79, 0.85)	0.93 (0.84, 1.00)	0.11 (0.07, 0.14)	0.57 (0.56, 0.58)	0.64 (0.53, 0.74)	0.07 (0.05, 0.08)
	40–49	0.79 (0.59, 0.99)	0.91 (0.79, 1.00)	0.12 (0.07, 0.16)	0.67 (0.57, 0.76)	0.73 (0.63, 0.82)	0.06 (0.05, 0.07)
	50–59	0.70 (0.43, 0.96)	0.83 (0.59, 1.00)	0.13 (-0.14, 0.41)	0.70 (0.60, 0.80)	0.79 (0.70, 0.88)	0.09 (0.07, 1.00)
	60+	0.68 (0.44, 0.93)	0.87 (0.71, 1.00)	0.19 (0.10, 0.27)	0.71 (0.60, 0.82)	0.75 (0.63, 0.86)	0.04 (0.02, 0.05)
	*p-value*^*c*^	*< 0*.*001*	*0*.*005*	-	*< 0*.*001*	*< 0*.*001*	-
Age at first sexual intercourse	< 18	0.76 (0.53, 0.98)	0.90 (0.77, 1.00)	0.15 (0.10, 0.20)	0.69 (0.59, 0.78)	0.73 (0.64, 0.82)	0.04 (0.03, 0.06)
	18+	0.79 (0.60, 0.98)	0.90 (0.78, 1.00)	0.12 (0.07, 0.17)	0.62 (0.51, 0.72)	0.70 (0.60, 0.80)	0.08 (0.07, 0.09)
	*p-value*	*0*.*848*	*0*.*996*	-	*0*.*012*	*0*.*200*	-
Number of lifetime sexual partners	0–1	0.74 (0.52, 0.96)	0.90 (0.77, 1.00)	0.16 (0.10, 0.22)	0.68 (0.58, 0.77)	0.72 (0.62, 0.81)	0.04 (0.03, 0.05)
	2–3	0.80 (0.62, 0.99)	0.90 (0.78, 1.00)	0.10 (0.06, 0.15)	0.60 (0.50, 0.70)	0.70 (0.60, 0.80)	0.10 (0.08, 0.11)
	4+	0.80 (0.59, 1.00)	0.91 (0.80, 1.00)	0.11 (-0.16, 0.39)	0.63 (0.51, 0.75)	0.72(0.61, 0.84)	0.09 (0.06, 0.11)
	*p-value*^*c*^	*0*.*096*	*0*.*769*	-	*<0*.*001*	*0*.*110*	-
History of STD	No	0.77 (0.57, 0.98)	0.90 (0.78, 1.00)	0.13 (0.10, 0.17)	0.65 (0.56, 0.75)	0.72 (0.62, 0.81)	0.06 (0.06, 0.07)
	Yes	0.77 (0.58, 0.97)	0.89 (0.75, 1.00)	0.12 (0.00, 0.23)	0.59 (0.47, 0.72)	0.66 (0.54, 0.78)	0.07 (0.05, 0.09)
	*p-value*	*0*.*540*	*0*.*910*	-	*< 0*.*001*	*0*.*093*	-
Parity	0–1	-	-	-	0.75 (0.64, 0.85)	0.79 (0.69, 0.89)	0.04 (0.03, 0.06)
	2–5	0.81(0.64, 0.99)	0.92 (0.82, 1.00)	0.11 (0.06, 0.16)	0.58 (0.47, 0.68)	0.64 (0.54, 0.74)	0.06 (0.05, 0.08)
	6–9	0.77 (0.57, 0.98)	0.90 (0.75, 1.00)	0.12 (0.08, 0.17)	0.67 (0.57, 0.76)	0.74 (0.65, 0.83)	0.07 (0.06, 0.08)
	10+	0.68 (0.43, 0.93)	0.89 (0.76, 1.00)	0.21 (0.09, 0.34)	0.67 (0.55, 0.78)	0.71 (0.59, 0.82)	0.04 (0.02, 0.06)
	*p-value*^*c*^	*0*.*014*	*0*.*269*	-	*0*.*036*	*0*.*003*	-
Menopausal status	Pre	0.81 (0.63, 0.99)	0.92 (0.81, 1.00)	0.11 (0.08, 0.14)	0.60 (0.50, 0.70)	0.67 (0.57, 0.77)	0.07 (0.06, 0.08)
	Post	0.66 (0.41, 0.92)	0.85 (0.66, 1.00)	0.19 (0.11, 0.27)	0.73 (0.63, 0.83)	0.79 (0.70, 0.88)	0.06 (0.05, 0.07)
	*p-value*	*0*.*421*	*0*.*621*	-	*< 0*.*001*	*< 0*.*001*	-
Healthcare provider	Medical doctor	0.78 (0.59, 0.98)	0.90 (0.76, 1.00)	0.11 (0.08, 0.15)	0.60 (0.50, 0.71)	0.71 (0.61, 0.80)	0.10 (0.09, 0.11)
	Other provider	0.72 (0.48, 0.96)	0.91 (0.79, 1.00)	0.19 (0.09, 0.30)	0.71 (0.61, 0.81)	0.72 (0.61, 0.82)	0.01 (0.00, 0.02)
	*p-value*	*0*.*729*	*0*.*931*	-	*< 0*.*001*	*0*.*644*	-
History of Pap test	Never	0.77 (0.57, 0.98)	0.90 (0.78, 1.00)	0.13 (0.09, 0.16)	0.64 (0.54, 0.74)	0.71 (0.61, 0.81)	0.07 (0.06, 0.08)
	Ever	-	-	-	0.86 (0.74, 0.97)	0.74 (0.59, 0.89)	-0.12 (-0.16, -0.08)
	*p-value*	-	-	-	*0*.*001*	*0*.*644*	-
HPV DNA	Negative	0.78 (0.59, 0.97)	0.84 (0.64, 1.00)	0.06 (0.02, 0.09)	0.65 (0.55, 0.75)	0.72 (0.63, 0.81)	0.07 (0.07, 0.08)
	Positive	0.78 (0.58, 0.99)	0.92 (0.82, 1.00)	0.14 (0.10, 0.18)	0.59 (0.44, 0.75)	0.58 (0.41, 0.74)	-0.01 (-0.05, 0.01)
	*p-value*	*0*.*617*	*0*.*577*	-	*< 0*.*001*	*< 0*.*001*	-
Cytology	NILM	0.68 (0.45, 0.90)	0.85 (0.68, 1.00)	0.17 (0.08, 0.27)	0.65 (0.56, 0.74)	0.71 (0.63, 0.80)	0.06 (0.06, 0.07)
	ASC-US	0.83 (0.66, 1.00)	0.87 (0.71, 1.00)	0.04 (-0.15, 0.23)	0.53 (0.35, 0.72)	0.69 (0.52, 0.86)	0.16 (0.10, 0.21)
	LSIL	0.81 (0.64, 0.98)	0.90 (0.77, 1.00)	0.09 (0.04, 0.13)	0.58 (0.40, 0.76)	0.65 (0.47, 0.83)	0.07 (0.01, 0.13)
	HSIL	0.80 (0.59, 1.00)	0.96 (0.89, 1.00)	0.16 (0.11, 0.21)	0.61 (0.34, 0.87)	0.44 (0.15, 0.72)	-0.17 (-0.32, -0.02)
	Cancer	0.79 (0.56, 1.00)	0.94 (0.84, 1.00)	0.15 (0.09, 0.21)	0.55 (0.23, 0.88)	0.44 (0.08, 0.80)	-0.11 (-0.18, -0.04)
	*p-value*^*c*^	*0*.*137*	*< 0*.*001*	-	*< 0*.*001*	*< 0*.*001*	-

Abbreviations: NILM: Negative for Intraepithelial Lesion or Malignancy; ASC-US: Atypical Cells of Undetermined Significance; LSIL: Low grade Squamous Intraepithelial Lesion; HSIL: High grade Squamous Intraepithelial Lesion; HPV DNA: Human Papillomavirus DNA test; STD: Sexually Transmitted Diseases; VIA: Visual Inspection with Acetic acid; VILI: Visual Inspection with Lugol’s Iodine.

^a^ Model-based sensitivity and specificity. Colposcopy with biopsy was used to define disease (CIN2+).

^b^ Difference between physician and nurse.

^c^ Non-parametric trend test p-value for characteristic with ordered categories. Other p-values are from z tests for binary characteristics.

**Table 3:** Determinants of VILI sensitivity and specificity performed by nurse and physician: the Congo Community-Based Screening Study.

Variable	Categories	VILI Sensitivity (95% CI)^a^			VILI Specificity (95% CI)^a^		
		Nurse	Physician	Difference^b^	Nurse	Physician	Difference^b^
Age (Years)	30–39	0.77 (0.57, 0.97)	0.93 (0.83, 1.00)	0.16 (0.11, 0.21)	0.72 (0.62, 0.81)	0.72 (0.62, 0.82)	0.00 (-0.01, 0.01)
	40–49	0.78 (0.58, 0.99)	0.92 (0.81, 1.00)	0.14 (0.08, 0.20)	0.75 (0.66, 0.84)	0.76 (0.67, 0.85)	0.01 (0.00, 0.02)
	50–59	0.67 (0.38, 0.96)	0.86 (0.64, 1.00)	0.19 (-0.03, 0.41)	0.79 (0.70, 0.87)	0.82 (0.73, 0.90)	0.03 (0.02, 0.04)
	60+	0.67 (0.42, 0.92)	0.85 (0.67, 1.00)	0.18 (0.09, 0.27)	0.78 (0.67, 0.88)	0.81 (0.70, 0.92)	0.03 (0.02, 0.04)
	*p-value*^*c*^	*0*.*018*	*0*.*006*	-	*< 0*.*001*	*< 0*.*001*	-
Age at first sexual intercourse	< 18	0.72 (0.48, 0.96)	0.91 (0.79, 1.00)	0.19 (0.13, 0.25)	0.78 (0.69, 0.87)	0.77 (0.68, 0.87)	-0.01 (-0.01, 0.00)
	18+	0.76 (0.55, 0.96)	0.90 (0.77, 1.00)	0.14 (0.10, 0.19)	0.73 (0.63, 0.82)	0.75 (0.66, 0.85)	0.02 (0.02, 0.03)
	*p-value*	*0*.*807*	*0*.*973*	*-*	*0*.*030*	*0*.*412*	-
Number of lifetime sexual partners	0–1	0.72 (0.49, 0.95)	0.90 (0.77, 1.00)	0.18 (0.12, 0.24)	0.76 (0.67, 0.84)	0.77 (0.68, 0.86)	0.01 (0.00, 0.02)
	2–3	0.76 (0.55, 0.97)	0.91 (0.79, 1.00)	0.15 (0.10, 0.20)	0.73 (0.64, 0.82)	0.75 (0.66, 0.84)	0.02 (0.01, 0.03)
	4+	0.78 (0.58, 0.99)	0.90 (0.77, 1.00)	0.12 (-0.26, 0.50)	0.75 (0.65, 0.86)	0.77 (0.66, 0.88)	0.02 (0.00, 0.03)
	*p-value*^*c*^	*0*.*227*	*0*.*896*	*-*	*0*.*006*	*0*.*026*	-
History of STD	No	0.74 (0.51, 0.96)	0.91 (0.78, 1.00)	0.17 (0.13, 0.21)	0.76 (0.67, 0.85)	0.77 (0.68, 0.86)	0.01 (0.00, 0.01)
	Yes	0.77 (0.57, 0.97)	0.89 (0.76, 1.00)	0.12 (0.02, 0.23)	0.67 (0.55, 0.79)	0.72 (0.60, 0.84)	0.05 (0.03, 0.07)
	*p-value*	*0*.*883*	*0*.*930*	*-*	*0*.*007*	*0*.*133*	-
Parity	0–1	-	-	-	0.85 (0.77, 0.93)	0.82 (0.72, 0.92)	-0.03 (-0.05, -0.02)
	2–5	0.79 (0.60, 0.98)	0.91 (0.80, 1.00)	0.12 (0.06, 0.19)	0.70 (0.60, 0.79)	0.73 (0.63, 0.82)	0.03 (0.02, 0.04)
	6–9	0.73 (0.50, 0.96)	0.90 (0.78, 1.00)	0.17 (0.13, 0.22)	0.76 (0.68, 0.85)	0.77 (0.68, 0.86)	0.01 (0.00, 0.01)
	10+	0.67 (0.43, 0.91)	0.87 (0.72, 1.00)	0.20 (0.01, 0.39)	0.73 (0.62, 0.84)	0.78 (0.68, 0.89)	0.05 (0.03, 0.07)
	*p-value*^*c*^	*0*.*034*	*0*.*256*	*-*	*0*.*004*	*0*.*003*	-
Menopausal status	Pre	0.77 (0.57, 0.97)	0.92 (0.82, 1.00)	0.15 (0.12, 0.19)	0.72 (0.63, 0.81)	0.73 (0.63, 0.82)	0.01 (0.00, 0.01)
	Post	0.65 (0.39, 0.91)	0.84 (0.64, 1.00)	0.19 (0.11, 0.27)	0.79 (0.70, 0.88)	0.83 (0.74, 0.91)	0.03 (0.03, 0.04)
	*p-value*	*0*.*546*	*0*.*557*	*-*	*0*.*003*	*< 0*.*001*	-
Healthcare provider	Medical doctor	0.73 (0.51, 0.95)	0.90 (0.77, 1.00)	0.17 (0.12, 0.21)	0.73 (0.64, 0.82)	0.76 (0.67, 0.85)	0.03 (0.03, 0.04)
	Other provider	0.74 (0.52, 0.96)	0.90 (0.77, 1.00)	0.16 (0.06, 0.27)	0.78 (0.69, 0.87)	0.76 (0.66, 0.86)	-0.02 (-0.02, 0.00)
	*p-value*	*0*.*852*	*0*.*826*	*-*	*< 0*.*001*	*0*.*421*	-
History of Pap test	Never	0.74 (0.52, 0.96)	0.90 (0.78, 1.00)	0.16 (0.12, 0.20)	0.74 (0.65, 0.83)	0.76 (0.67, 0.85)	0.02 (0.01, 0.02)
	Ever	-	-	-	0.94 (0.86, 1.00)	0.82 (0.69, 0.95)	-0.12 (-0.15, -0.09)
	*p-value*	*N/A*	*N/A*	*-*	*0*.*002*	*0*.*339*	-
HPV DNA	Negative	0.68 (0.45, 0.91)	0.84 (0.66, 1.00)	0.16 (0.12, 0.20)	0.75 (0.67, 0.83)	0.78 (0.69, 0.86)	0.03 (0.02, 0.03)
	Positive	0.75 (0.53, 0.97)	0.92 (0.81, 1.00)	0.17 (0.12, 0.21)	0.71 (0.57, 0.85)	0.64 (0.47, 0.80)	-0.08 (-0.10, -0.05)
	*p-value*	*0*.*775*	*0*.*622*	*-*	*0*.*31*	*< 0*.*001*	-
Cytology	NILM	0.62 (0.38, 0.86)	0.83 (0.64, 1.00)	0.21 (0.14, 0.27)	0.75 (0.67, 0.83)	0.77 (0.69, 0.85)	0.02 (0.01, 0.02)
	ASC-US	0.68 (0.42, 0.94)	0.88 (0.73, 1.00)	0.20 (0.10, 0.32)	0.75 (0.59, 0.91)	0.74 (0.57, 0.90)	-0.01 (-0.05, 0.03)
	LSIL	0.76 (0.57, 0.96)	0.89 (0.76, 1.00)	0.13 (0.08, 0.17)	0.66 (0.49, 0.83)	0.70 (0.52, 0.87)	0.04 (0.01, 0.07)
	HSIL	0.80 (0.60, 1.00)	0.97 (0.91, 1.00)	0.17 (0.11, 0.22)	0.62 (0.35, 0.89)	0.44 (0.15, 0.74)	-0.17 (-0.32, -0.04)
	Cancer	0.80 (0.58, 1.00)	0.95 (0.86, 1.00)	0.15 (0.10, 0.21)	0.56 (0.24, 0.88)	0.45 (0.08, 0.81)	-0.11 (-0.17, -0.05)
	*p-value*^*c*^	*< 0*.*001*	*< 0*.*001*	*-*	*< 0*.*001*	*< 0*.*001*	-

Abbreviations: NILM: Negative for Intraepithelial Lesion or Malignancy; ASC-US: Atypical Cells of Undetermined Significance; LSIL: Low grade Squamous Intraepithelial Lesion; HSIL: High grade Squamous Intraepithelial Lesion; HPV DNA: Human Papillomavirus DNA test; STD: Sexually Transmitted Diseases; VIA: Visual Inspection with Acetic acid; VILI: Visual Inspection with Lugol’s Iodine

^a^ Model-based sensitivity and specificity. Colposcopy with biopsy was used to define disease (CIN2+).

^b^ Difference between physician and nurse.

^c^ Non-parametric trend test p-value for characteristic with ordered categories. Other p-values are from z te.

In general, there was a significant difference in performance between the physician and the nurse. For VIA, the sensitivity of the physician’s assessment was 13% higher in absolute terms than the nurse’s (95% CI: 0.09–0.16). The physician scoring was also more specific than the nurse’s (difference = 6%; 95% CI: 0.06–0.07). The same was observed for VILI: difference in sensitivity: 16% (95%CI: 0.12, 0.20); difference in specificity: 1% (95% CI: 0.01, 0.02), favoring the physician assessment. The differences observed across most characteristics show that the physician tended to be more accurate than nurses, with few exceptions.

We used the Coughlin’s model approach [17] to estimate sensitivity and specificity of VIA and VILI cervical cancer screening tests as a function of potential influences in performance and to identify potential characteristics that would lead to inadequate screening performance in field work conditions. We found that younger age, higher parity, pre-menopausal status, HPV positivity, and Pap cytology test results can be important influences on the performance of VIA and VILI tests in this study population. We found that a physician’s examination affords higher sensitivity and specificity for these visual screening tests than that performed by nurses, across most predictors.

Although previous studies have investigated predictors of VIA positivity [9–11], they focused on estimating odds ratios, an approach that is better suited for evaluation of effectiveness rather than the diagnostic accuracy of screening tests [19]. Hence, it may be difficult to compare our findings with previous reports, especially for VILI which has been less extensively studied. Nonetheless, an effect of age on VIA positivity rate was repeatedly observed [5,9,10,20]. We found that sensitivity of VIA and VILI decreased while specificity increased as age increased. Women with higher parity were reported to have an increased risk of a positive VIA in some studies [10,21], but not all [8]. VIA positivity was not found to be associated with cytology results [11] or with HPV positivity [10]. Conversely, women who tested positive for HPV were significantly more likely to have a positive VIA (15%, 95% CI: 12.9–17.2) than HPV negative women (6.3%, 95% CI: 5.7–6.9) [9]. Moreover, a CIN2^+^ diagnosis and HPV positivity with a higher viral load were found to be determinants of VIA positivity [9].

Our results show variation in nurse- and physician-based VIA and VILI positivity rates, that were also higher, especially for VIA, than previously reported figures [9–11]. Variability in VIA positivity across studies could be attributed to several factors, such as the skill and experience of the health care provider performing the test, variation in the light source, procedure for preparation of 4–5% acetic acid solution and its storage, underlying prevalence of sexually transmitted diseases or cervical disease, and the choice of gold standard or non-uniformity of the gold standard for disease definition [21,22].

Studies of inter-rater agreement on the performance of visual inspection techniques for precancerous lesions of cervical cancer are very limited, but good concordance is generally reported. Moderate to substantial agreement for VIA performance was found between test providers [6,23,24], with no adjustment for patient characteristics. A VIA by nurse compared to that performed by physician had higher sensitivity (100% vs. 87.5%) and lower specificity (53% vs. 63%), but no statistically significant differences in the diagnostic accuracy of VIA were found between nurse and physician [6]. Another study found higher sensitivity (88.9% vs. 80.0%) and specificity (69.8% vs. 54.9%) for VIA by physician compared to VIA by nurse, but the differences were not statistically tested [23]. An overall agreement of 66.7% [Kappa value = 0.57 (95% CI: 0.48, 0.66)] was reported for three independent raters assessing cervical photographs after acetic acid wash, but sensitivity and specificity were not estimated [24]. In the current study, the overall sensitivity (VIA: 90.3% vs. 77.4%; VILI: 90.3% vs. 74.2%) and specificity (VIA: 71.0% vs. 64.5%; VILI: 76.2% vs. 74.7%) were higher for the physician compared with nurses as examiners. The significant differences in the sensitivity and specificity observed between nurse and physician could be due to adjustment for other risk factors in the logistic regression models.

Subjectivity in the interpretation of visual test results by different test providers and high false positive rates are acknowledged limitations of visual inspection screening tests [6,12,25]. In our study, the false positive rates of VIA and VILI differed significantly between nurse and physician (VIA: 35.4% vs. 29%; VILI: 25.3% vs. 23.8%). Other limitations that need to be underscored include the cross-sectional nature of the study, and our inability to explore inter-nurse variability as a function of field experience with VIA and VILI techniques, as this information was not available.

The main strength of our study is that it is the first report to use a statistical approach (the Coughlin model) to calculate sensitivity and specificity of VIA and VILI tests while accounting for potential patient characteristics that can influence test accuracy. Moreover, ours is the first to investigate whether the accuracy of VIA and VILI tests depends on the test provider, while simultaneously controlling for other patient characteristics. Other strengths of this study include its population-based design, high response rate, the fact that cytopathologists and pathologists were blinded to the results of the screening tests, and that all women underwent colposcopy, thus minimizing the likelihood of verification bias which arises when the only individuals to undergo the reference standard test for verification of disease are those who have screened positive [26,27].

## Conclusions

The diagnostic accuracy of VIA and VILI tests performed by nurse and physician were significantly related to being younger, having high parity, pre-menopausal status, HPV positivity, and cytology with an ASC-US cut-off status. Our results suggest that improved training of healthcare providers (prior to their being deployed as screeners) to take these determinants into account will enhance the performance of VIA and VILI in detecting cervical precancerous lesions among women in low-resource settings. To improve their diagnostic accuracy, more training of the nurse on case definition and interpretation of definite aceto-white cervical epithelium of VIA test is also warranted.

## Supporting Information

S1 Table An anonymized version of the non-imputed dataset available in an excel format. (XLSX)

## Abbreviations

ASC-US: Atypical squamous cells of undetermined significance
CI: Confidence interval
CIN: Cervical intraepithelial neoplasia
HSIL: High-grade squamous intraepithelial lesion
LSIL: Low-grade squamous intraepithelial lesion
NILM: Negative for intraepithelial lesions or malignancy
VIA: Visual inspection with acetic acid
VILI: Visual inspection with Lugol’s iodine
